# Eisosome disruption by noncoding RNA deletion increases protein secretion in yeast

**DOI:** 10.1093/pnasnexus/pgac241

**Published:** 2022-10-26

**Authors:** Matthew Wenjie Feng, Daniela Delneri, Catherine B Millar, Raymond T O'Keefe

**Affiliations:** Division of Evolution, Infection and Genomics, Faculty of Biology, Medicine and Health, The University of Manchester, Oxford Road, Manchester M13 9PT, UK; Division of Evolution, Infection and Genomics, Faculty of Biology, Medicine and Health, The University of Manchester, Oxford Road, Manchester M13 9PT, UK; Manchester Institute of Biotechnology, Faculty of Biology Medicine and Health, The University of Manchester, 131 Princess street, Manchester, M1 7DN, UK; Division of Evolution, Infection and Genomics, Faculty of Biology, Medicine and Health, The University of Manchester, Oxford Road, Manchester M13 9PT, UK; Division of Evolution, Infection and Genomics, Faculty of Biology, Medicine and Health, The University of Manchester, Oxford Road, Manchester M13 9PT, UK

**Keywords:** noncoding RNA, PIL1, eisosome membrane compartment (EMC), protein secretion, *Saccharomyces cerevisiae*

## Abstract

Noncoding RNAs (ncRNAs) regulate many aspects of gene expression. We investigated how ncRNAs affected protein secretion in yeast by large-scale screening for improved endogenous invertase secretion in ncRNA deletion strains with deletion of stable unannotated transcripts (SUTs), cryptic unstable transcripts (CUTs), tRNAs, or snRNAs. We identified three candidate ncRNAs, SUT418, SUT390, and SUT125, that improved endogenous invertase secretion when deleted. As SUTs can affect expression of nearby genes, we quantified adjacent gene transcription and found that the *PIL1* gene was down-regulated in the SUT125 deletion strain. Pil1 is a core component of eisosomes, nonmobile invaginations found throughout the plasma membrane. *PIL1* knockout alone, or in combination with eisosome components *LSP1* or *SUR7*, resulted in further increased secretion of invertase. Secretion of heterologous GFP was also increased upon *PIL1* deletion, but this increase was signal sequence dependent. To reveal the potential for increased biopharmaceutical production, secretion of monoclonal antibody Pexelizumab scFv peptide was increased by *PIL1* deletion. Global analysis of secreted proteins revealed that approximately 20% of secreted proteins, especially serine-enriched secreted proteins, including invertase, were increased upon eisosome disruption. Eisosomes are enriched with APC transporters and sphingolipids, which are essential components for secretory vesicle formation and protein sorting. Sphingolipid and serine biosynthesis pathways were up-regulated upon *PIL1* deletion. We propose that increased secretion of endogenous and heterologous proteins upon *PIL1* deletion resulted from sphingolipid redistribution in the plasma membrane and up-regulated sphingolipid biosynthesis. Overall, a new pathway to improve protein secretion in yeast via eisosome disruption has been identified.

Significance Statement Eukaryotic genomes are pervasively transcribed, generating a plethora of noncoding RNAs (ncRNAs). How ncRNAs affect protein secretion remains unknown. We show that deletion of the ncRNA SUT125 increases endogenous invertase secretion by down-regulating the adjacent gene *PIL1*. Pil1 is a core component of eisosomes, which act as plasma membrane reservoirs and sequester sphingolipids. Direct eisosome disruption revealed a further increase in invertase secretion as well as increased heterologous GFP and antibody secretion. We propose that disruption of eisosomes redistributes sphingolipids and transmembrane proteins Sur7 and Nce102, activating sphingolipid signaling and biosynthesis, accelerating lipid and protein trafficking and membrane fusion, and increasing exocytosis. Our study reveals that the eisosome is a promising target for increasing protein secretion and biopharmaceutical production in yeast.

## Introduction

Heterologous protein expression involves the introduction of foreign genes into a broad range of organisms to produce recombinant proteins. Recombinant proteins are widely applied in the fields/areas of biochemical, biopharmaceutical, bioenergy, industrial fermentation, and biomaterials. Budding yeast is one of the main organisms used for heterologous protein expression because of its unique characteristics. In comparison to bacteria, yeast have post-translational modification (PTMs) such as methylation, phosphorylation, and glycosylation. These PTMs are critical to the function of certain human proteins, and the lack of such PTMs in bacteria make bacteria less versatile than yeast. Compared with mammalian cells, yeast have the characteristics of rapid growth, economical growth conditions, and ease of genetic manipulation, which makes it an ideal organism to develop strategies to improve the expression of heterologous proteins and to produce proteins on an industrial scale (1).

To manipulate expression of a protein of interest in yeast, attention has been given to the protein secretion pathway. Modifications of secretory signal peptides improved secretion of heterologous proteins (2, 3). The engineering of the unfolded protein response, stress tolerance, protein trafficking, glycosylation process, and protein degradation process also improved the secretion of heterologous proteins to different levels, but often these improvements were only observed for specific proteins (4–6). Genome-wide screenings for target genes improving protein secretion have identified novel targets with functions in cellular metabolism and the cell cycle (7). The expression of heterologous proteins in yeast was also enhanced by overexpression of essential genes involved in the protein secretion pathway or by overexpressing the heterologous protein itself. This overexpression, however, imposed a burden on the host cell, causing the activation of endoplasmic reticulum-associated degradation (ERAD) pathway and resulting in cellular growth defects (8–12). Moreover, a genome-wide profiling study with the yeast heterozygote protein deletion collection has shown that protein secretion is the cellular category affected the most by gene dosage and changes in expression will have a large effect on cell homeostasis (13). Hence, achieving a fine tuning of expression for these genes by manipulating noncoding RNA (ncRNA) regulatory elements would be desirable.

Recent research has revealed the importance of ncRNAs in the regulation of gene expression. In yeast, subsets of ncRNAs known as Stable Unannotated Transcripts (SUTs) and Cryptic Unstable Transcripts (CUTs) have been identified (14–17). These SUTs and CUTs are suspected to have transcriptional and post-transcriptional effects on their nearby genes (18, 19). Some SUTs and CUTs also have global *in trans* effects on the protein regulatory network (20–23). In particular, the essential gene *SEC4* encoding a Rab family GTPase that is involved in Golgi to plasma membrane transport, vesicle fusion, and protein secretion has been shown to be regulated by the nearby SUT527 (20). Therefore, manipulating ncRNAs may be another important way to increase heterologous protein production in yeast.

Eisosomes were previously proposed to be the static sites of endocytosis (24), but have recently been recognized as static, furrow-like, invaginations of the plasma membrane (25–28). In response to acute mechanical or osmotic stress at the plasma membrane, eisosome invaginations are disrupted to provide additional membrane reservoirs (29). In addition, eisosomes are reported to contain a large amount of sphingolipids (30, 31) and ergosterol (26, 27) that function in signal transduction and protein trafficking (32). Disruption of eisosomes, caused by plasma membrane stress, induces sphingolipid signaling and biosynthesis via the relocalization of Nce102 and Slm1/2 from eisosomes to membrane compartments containing Pma1p (MCPs) and membrane compartments containing Tor2p (MCTs) followed by the activation of rapamycin complex 2 (TORC2) and Pkh1/2-Ypk1/2 signaling pathways (30, 31, 33, 34). Sphingolipids are essential for secretory vesicle formation and protein sorting, thus maintaining homeostasis and ensuring efficient delivery of secreted proteins in the early secretory pathway (35–40).

Here, we used the *Saccharomyces cerevisiae* ncRNA deletion strain collection to screen for strains with increased invertase secretion. We identified the top three ncRNA deletion strains with increased invertase secretion: SUT418Δ, SUT390Δ, and SUT125Δ. To investigate how deletion of these ncRNAs affected invertase secretion, we measured the gene expression adjacent to these ncRNAs, and found that *PIL1*, a core component of eisosomes in the plasma membrane, had decreased expression in the SUT125 deletion strain. Deleting *PIL1* directly caused increased secretion of endogenous invertase, heterologous GFP, and the biopharmaceutical monoclonal antibody Pexelizumab (Pex) scFv peptide. Furthermore, global analysis of secretion revealed the abundance of approximately 20% of yeast secreted proteins, especially serine-enriched secreted proteins, including invertase, was significantly increased upon *PIL1* deletion. We propose that disruption of eisosomes causes a global change in lipid homeostasis, contributing to secretory vesicle formation and protein sorting, which subsequently increases protein secretion.

## Results

### High-throughput screening reveals increased invertase secretion in SUT418, SUT390, and SUT125 ncRNA deletion strains

**Fig. 1.**
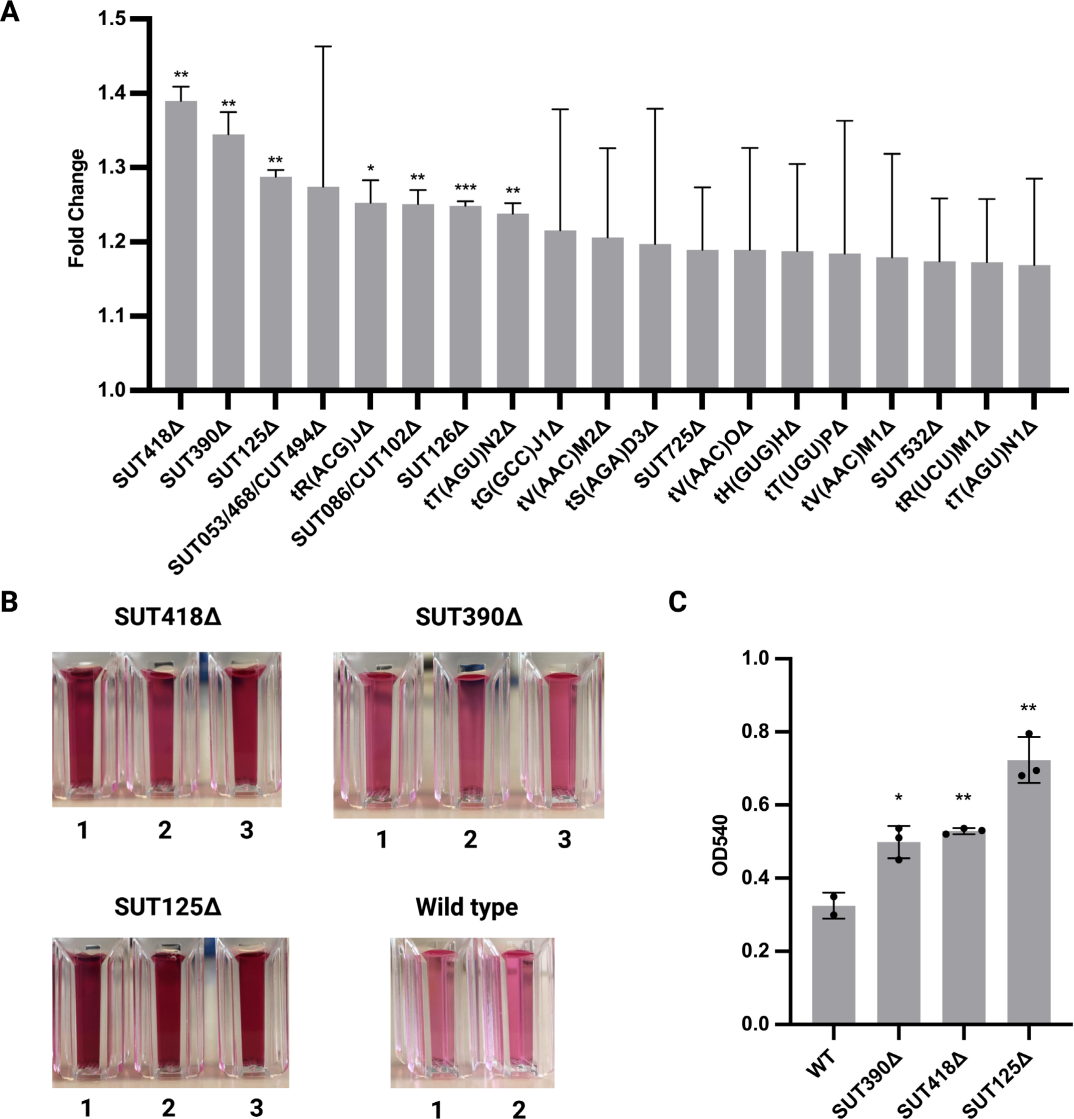
High throughput invertase assay screening identified SUT418, SUT390, or SUT125 deletion as having increased invertase secretion amongst 434 ncRNA deletion strains. (A) Top 20 candidates screened from high throughput invertase assay. Changes in invertase secretion for each ncRNA deletion strain are displayed in fold changes (ratio of ncRNA deletion mutant to wild-type). Data were derived from three biological replicates for each strain. ***P = 0.0008, **P < 0.00099, *P = 0.0188. (B) Individual invertase activity assay for secreted invertase of the SUT418, SUT390, and SUT125 deletion strains compared to the wild-type strain. *n* = 3 for SUT418, SUT390, SUT125, *n* = 2 for wild-type. (B) Quantification of invertase activity assay of the SUT418, SUT390, and SUT125 deletion strains compared to the wild-type strain. The invertase activity in each deletion strain was quantitated by the change of absorbance in OD_540_ compared to the wild-type strain. Error bars represent SDs and dots represent biological replicates in each deletion strain. Unpaired parametric t test was used. **P < 0.0042 and *P = 0.0194.

To identify ncRNAs with the ability to influence protein secretion, we screened 434 ncRNA deletion strains, including SUTs, CUTs, tRNAs, and snRNAs for improved protein secretion (Table S1). The change in protein secretion was quantified by an invertase assay. Invertase, encoded by the *SUC2* gene, is an enzyme secreted into the yeast periplasm under low glucose conditions to convert extracellular sucrose as an alternative carbon source to glucose and fructose for cell use (Figure S1A) (41, 42). Through the invertase assay, the invertase activity can be quantitated by the amount of glucose converted from sucrose and visualized using a color reaction (Figure S1B). The invertase secretion in each ncRNA deletion strain was assayed and compared to the wild-type, and the top 20 candidates with more than 20% increase in fold change were identified (Fig. 1A). Furthermore, the top three ncRNA deletion strains for invertase secretion (SUT418, SUT390, and SUT125) from the high throughput assay were assayed individually for invertase activity to confirm the results (Fig. 1B and C). Both assays revealed that invertase secretion in the SUT418, SUT390, and SUT125 deletion strains was increased in a range of 1.3- to 2-fold change.

### *PIL1*, the core component of eisosomes, is down-regulated by SUT125 deletion

**Fig. 2.**
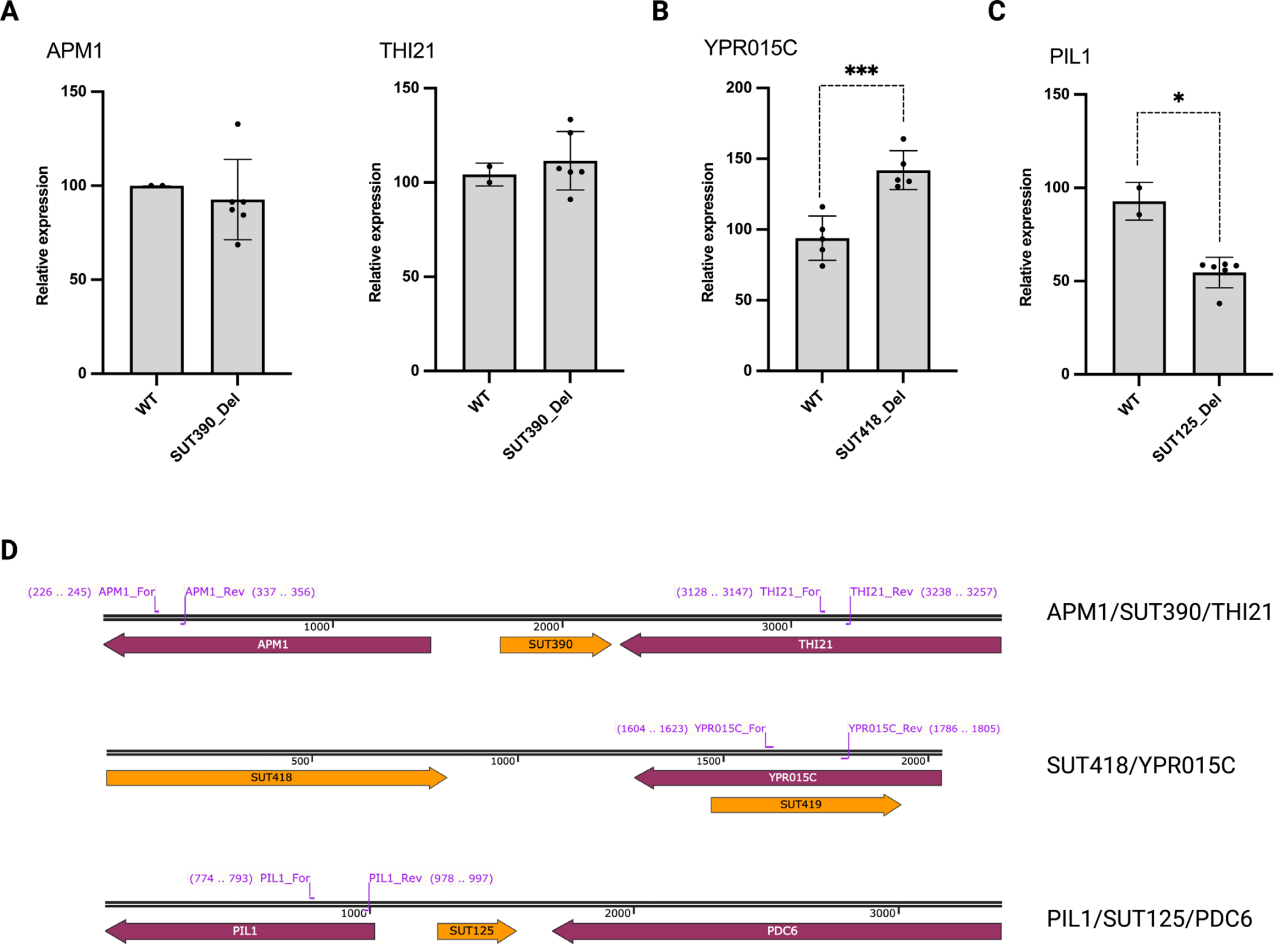
Effects of deleting ncRNAs on adjacent protein-coding genes. (A) Quantification of *APM1* and *THI21* transcript level in the SUT390 deletion strain compared to the wild-type strain. *N* = 6 for SUT390, *N* = 2 for wild-type. (B) Quantification of *YPR015C* transcript level in the SUT418 deletion strain compared to the wild-type strain. *N* = 5 for SUT418△, *N* = 5 for wild-type. (C) Quantification of *PIL1* transcript level in the SUT125 deletion strain compared to the wild-type strain. *N* = 6 for SUT125△, *N* = 2 for wild-type. (D) Organization of SUT390, SUT418, SUT125 and the respective neighboring protein-coding genes in the yeast genome. Primer pairs indicate the region of each gene examined by RT-qPCR (purple). Unpaired parametric t test was used. *** *P* = 0.0009 and *P = 0.0378.

To determine whether deletion of SUT390, SUT418, or SUT125 affected expression of nearby protein coding genes to enhance protein secretion, RT-qPCR was carried out for the adjacent genes in the wild-type and respective deletion strains (Fig. 2). Both the SUT390 and SUT125 have an upstream protein coding gene with which they share the promoter region and are transcribed divergently. All three SUTs also have a downstream protein coding gene that is transcribed convergently, although in the case of SUT418 there are some intervening ncRNA transcripts (Fig. 2D). Specifically, the genes examined included *APM1* and *THI21* (adjacent to SUT390); *YPR015C* (adjacent to SUT418); *PDC6* and *PIL1* (adjacent to SUT125). *APM1* encodes an AP-1 complex subunit that is involved in clathrin-dependent Golgi protein sorting. The promoter region of *APM1* overlaps with the promoter region of SUT390, but qPCR revealed no change of *APM1* expression in the SUT390 deletion strain (Fig. 2A). *THI21* encodes HMP-phosphate kinase that is involved in thiamine biosynthesis. Engineering of cellular metabolism such as thiamine biosynthesis may be a potential way to increase protein production (7). However, no significant change was detected in the expression of *THI21* in the SUT390 deletion strain (Fig. 2A). *YPR015C* encodes a zinc finger transcription factor that regulates transcription. The expression of *YPR015C* increased by about 50% in the SUT418 deletion strain (Fig. 2B). *PDC6* encodes a decarboxylase that is involved in amino acid catabolism. It has already been reported that *PDC6* is up-regulated in the SUT125Δ background and *PDC6* deletion results in reduced fitness similar to that detected in the SUT125Δ strain (21). Whether *PDC6* is related to protein secretion remains to be explored. *PIL1* encodes a core component of eisosomes responsible for the formation of furrow-like invaginations in the plasma membrane. The expression of *PIL1* was down-regulated by 50% in the SUT125 deletion strain compared to the wild-type (Fig. 2C). This *PIL1* down-regulation may be the result of *PIL1* sharing a bidirectional promoter with SUT125 (Fig. 2D). The deletion of SUT125 along with the insertion of the KanMX cassette may decrease the transcriptional activity of the bidirectional promoter region, thereby suppressing the expression of *PIL1*. Overall, these data suggest a link between the eisosome and protein secretion which may be regulated via SUTs.

### Eisosome disruption increases invertase secretion

To confirm that down-regulation of *PIL1* expression and the disrupted eisosome subsequently benefits protein secretion, we deleted *PIL1* along with other components of the eisosome (Figure S2) and observed the changes in protein secretion. In addition to *PIL1*, there are two other components of the eisosome, *LSP1* and *SUR7*. Together with *PIL1, LSP1* forms the eisosome invaginations, a subcortical structure of plasma membrane, and *SUR7* has a putative function in anchoring the eisosome to the inner plasma membrane (24, 43). Since the eisosome may also indirectly affect endocytosis (44, 45), *RVS161* encoding a protein involved in the formation of endocytic vesicles at the plasma membrane was included. The invertase assay was used to determine the changes in protein secretion regulated by *PIL1, LSP1, SUR7*, or *RVS161*.

**Fig. 3.**
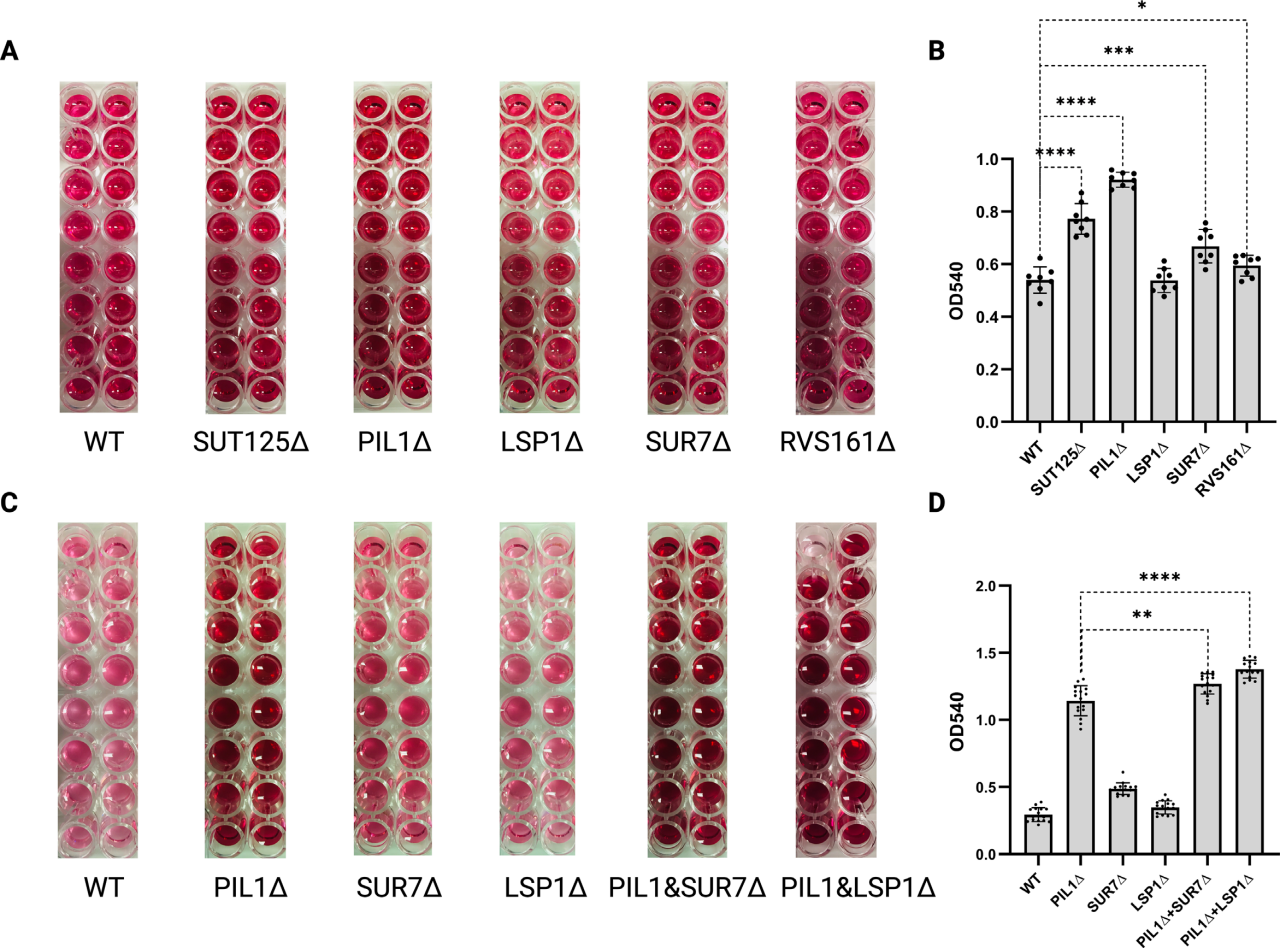
Changes in invertase secretion with single and double mutants of eisosome and endocytosis components. (A) Invertase activity assay for secreted invertase of SUT125, *PIL1, LSP1, SUR7*, and *RVS161* deletion strains compared to the wild-type strain. *n* = 16 for all strains. (B) Quantification of invertase activity assay of the SUT125, *PIL1, LSP1, SUR7*, and *RVS161* deletion strains compared to the wild-type strain. (C) Invertase activity assay for secreted invertase of *PIL1, LSP1, SUR7, PIL1/LSP1*, and*PIL1/SUR7* deletion strains compared to the wild-type strain. *n* = 16 for all strains. (D) Quantification of invertase activity assay of the *PIL1, LSP1, SUR7, PIL1&LSP1*, and *PIL1&SUR7* deletion strains compared to the wild-type strain. The invertase activity in each deletion strain was quantified by the change of absorbance in OD_540_ compared to the wild-type strain. Bars show averages of absorbances in each strain. Unpaired parametric t test was used. ****P < 0.0001, ***P = 0.0005, **P = 0.0026, and *P = 0.0285.

Deletion of *LSP1* had no effect on invertase secretion. In contrast, the deletion of *SUR7* and *RVS161* moderately increased invertase secretion. The deletion of *PIL1* increased the invertase secretion further, more than the SUT125 deletion in which *PIL1* was 50% down-regulated (Fig. 3A and B). Both the *PIL1/SUR7* and *PIL1/LSP1* double deletions revealed a moderate increase in invertase secretion compared to the *PIL1* single deletion (Fig. 3C and D). It has been reported that the deletion of PIL1 alone or with LSP1 causes structural disassembly of eisosomes which was confirmed by microscopy (24, 46). Therefore, the increased invertase secretion in the PIL1/LSP1 double deletion strain we suggest is due to the disruption of the eisosome. Taken together, these results reinforce the potential to increase protein secretion in yeast by disruption of the eisosome.

### Eisosome disruption increases GFP secretion

Previous studies have reported that increases in heterologous protein secretion tended to be protein specific or signal sequence specific (47). We next set out to understand whether disrupting the eisosome had any effect on the secretion of heterologous proteins and whether it was signal sequence specific. We constructed two GFP expression plasmids, each containing a different signal sequence, pre-Ost1 and pre-pro-α Factor (48), and individually integrated them into the genome of wild-type and *PIL1* deletion strains. Next, we verified the GFP copy number by a diagnostic PCR to ensure consistency between wild-type and *PIL1* deletion (Figure S3A and B). We further confirmed GFP mRNA levels by RT-qPCR (Figure S3C). To compare the GFP secretion level between the wild-type and *PIL1* deletion, only strains with a single copy of GFP were used for the downstream assays.

**Fig. 4.**
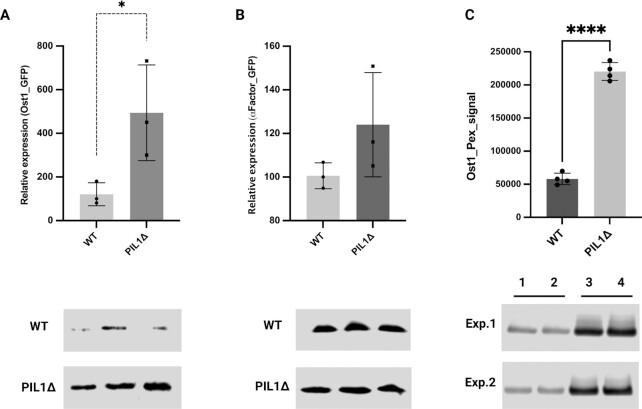
Difference in secretion of GFP with two different signal sequences and Pexelizumab (Pex) linked to a pre-Ost1 signal peptide between wild-type and *PIL1* deletion strains. (A) GFP secretion with pre-Ost1 signal peptide. (B) GFP secretion with pre-pro-α factor signal peptide. (C) Pexelizumab (Pex) scFv peptide secretion with pre-Ost1 signal peptide. 1 and 2 (3 and 4) were protein samples extracted from two individual colonies from the wt (*PIL1Δ*) strain. A total of two independent experiments (Experiment 1 and Experiment 2) were carried out. Upper panel quantitation, lower panel western blotting image. Images were quantified by LI-COR ImageStudio. Data were derived from three replicates. Unpaired parametric t test was used. *P = 0.0452 and ****P < 0.0001.

Western blotting was used to determine the GFP secretion level between wild-type and *PIL1* deletion. Cell cultures in log phase were normalized based on the OD_600_. Secreted proteins from the same amount of culture supernatant from wild-type or *PIL1* deletion strain cultures were separated by SDS-PAGE and GFP detected by western blot. When compared to the wild-type, GFP secretion was dramatically higher (3- to 7-fold) in the *PIL1Δ* strain containing GFP with the pre-Ost1 signal sequence (Fig. 4A). With the *PIL1Δ* strain containing GFP with the pre-pro-α factor signal sequence, GFP secretion was also higher (1- to 1.5-fold), but the difference compared to the wild-type was not statistically significant (Fig. 4B). Interestingly, the intracellular GFP level linked with a pre-Ost1 signal peptide was also higher in the *PIL1Δ* strain, and most of the intracellular GFP was the precursor protein (GFP linked with signal peptide; Figure S4). Taken together, these results indicated that disruption of the eisosome may increase the expression and secretion of heterologous proteins with specific signal sequences.

### Eisosome disruption increases Pexelizumab scFv peptide secretion

To reveal the potential of eisosome disruption in biopharmaceutical production, the gene encoding the monoclonal antibody Pexelizumab scFv peptide (Pex) was linked to a pre-Ost1 signal sequence and integrated into a wildtype and *PIL1Δ* strain. The mRNA levels varied between the wildtype and *PIL1Δ* integrated with Pex due to the strategy of plasmid integration (Figure S5). However, only colonies displaying the same expression levels of Pex were selected for downstream protein analysis. Secreted proteins were harvested and normalized from the culture supernatants. Western blotting revealed an approximately 4-fold increase of Pex secretion upon *PIL1* deletion (Fig. 4C). The increased secretion of both GFP and Pex indicated that the strategy of *PIL1* deletion/eisosome disruption combined with the use of the pre-Ost1 signal peptide may be a promising approach to increase the secretion of heterologous proteins in *S. cerevisiae*.

### Global effects of *PIL1* deletion on the yeast secretome

**Fig. 5.**
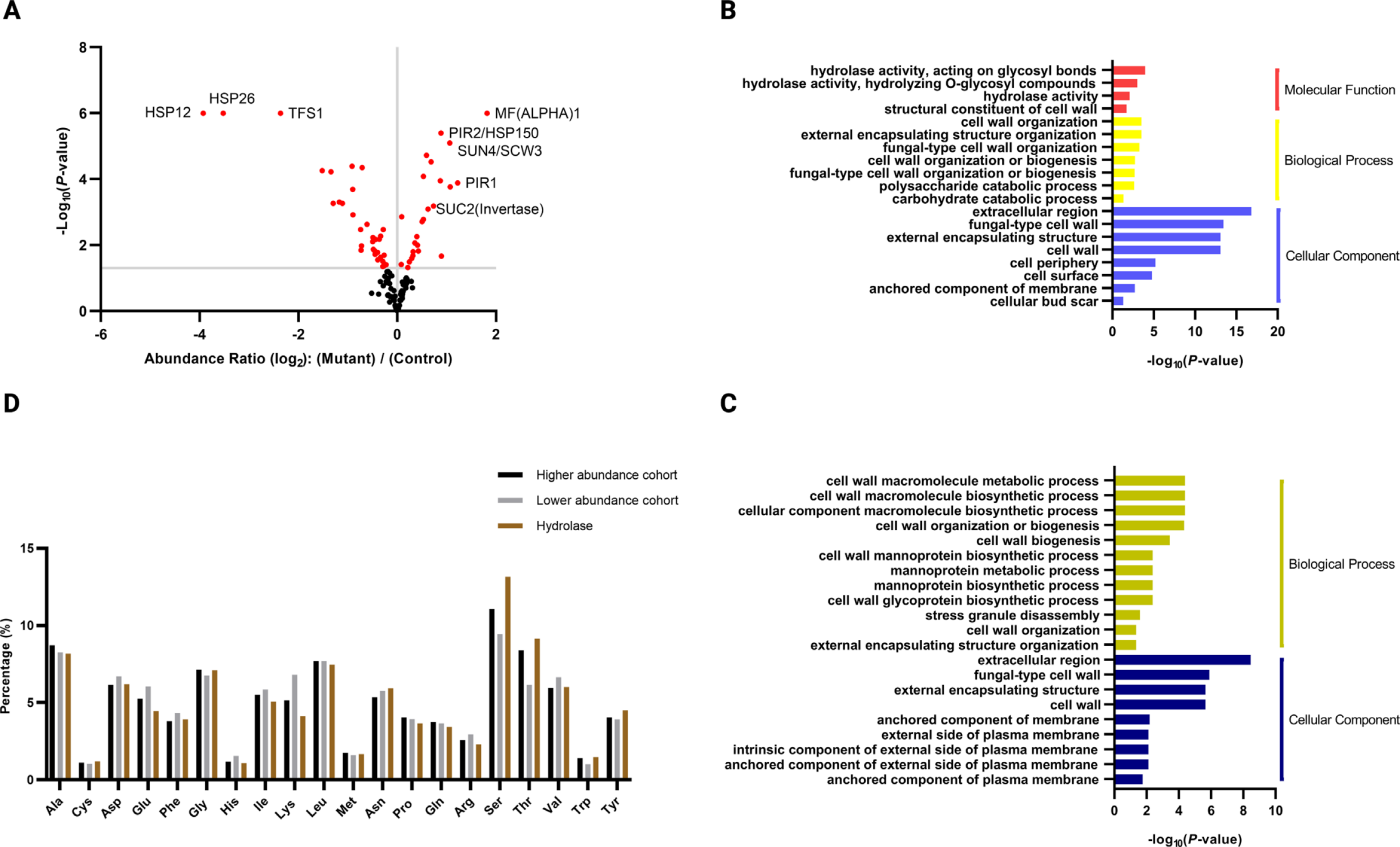
Global analysis of yeast secretome in the *PIL1* deletion strain by mass spectrometry. (A) Abundance ratio of quantified secreted proteins between the *PIL1* mutant and the wild-type. The ratio is presented in Log_2_ fold change. Red spots represent abundance ratios with *P*-value lower than 0.05. Some proteins with significant changes in secretion are annotated. (B) Gene ontology analysis of secreted proteins identified with higher abundance. (C) Gene ontology analysis of secreted proteins identified with lower abundance. (D) Percentages of amino acids in the protein sequence of higher and lower abundance cohorts and identified hydrolases. All data were derived from three SILAC experiments.

Since the effects of *PIL1* deletion on endogenous and heterologous proteins have been revealed, we suspected that eisosome disruption may have global effects on the yeast secretome. Thus, secretion from the *PIL1* deletion and wild-type strains was analyzed by liquid chromatography–tandem mass spectrometry (LC–MS/MS) using concentrated supernatant from the cell culture to reveal the global changes in the yeast secretome. To compare the secretome between the *PIL1* deletion mutant and the wild-type, stable isotope labeling by amino acids in cell culture (SILAC) was performed using the stable isotopes of arginine and lysine. A total of 142 secreted proteins were identified in three SILAC experiments, of which 123 were quantified. Signal peptides were present in 69 of 142 identified proteins. GPI anchors were present in 21 of 142 identified proteins (Table S2). Among the 123 quantified proteins, 59 had significantly different secretion levels (*P*-value < 0.05) when comparing the *PIL1* deletion strain with the wild-type (Fig. 5A). Invertase was detected with 1.4- to 1.7-fold change, which is consistent with the results from the invertase assay (Fig. 5A; Table S2). The secreted proteins with the highest or lowest abundances relative to wild-type were annotated (Fig. 5A). Proteins whose secretion were the most up-regulated were associated to cell mating (i.e. MFalpha1 and Sun4/Scw3) and cell wall stability (Pir1, Pir2/Hsp150, and Sun4/Scw3), whereas those with the most down-regulated secretion were mainly heat shock proteins (Hsp12 and Hsp26). Overall, 20.3% secreted proteins quantified by three separate SILAC experiments are more abundant, whereas 27.6% were less abundant, and 52.1% had no significant difference. The total protein content of the higher abundance cohort was increased by 5.77%, whereas the lower abundance cohort was decreased by 4.57% (Table S2). Gene ontology analysis revealed that proteins with changes in abundance were involved in cell wall structure, organization or biogenesis (Fig. 5B and C), with the higher abundant proteins characterized by hydrolase activities contributed mainly by invertase, glucosidase, glucanase, aspartic proteinase, and lysophospholipase (Fig. 5B, red). By analyzing the amino acid compositions of the secreted proteins, we found that the proportion of serine and threonine residues in the more abundant protein cohort was higher than in the lower abundance cohort (approximately 4%). Hydrolases had the highest proportion of serine and threonine, up to 9% to 13% (Fig. 5D); whereas in the amino acid compositions of the signal peptides, we found that the proportion of alanine residues displayed the main difference (approximately 5%) between the more abundance cohort and the less abundance cohort (Figure S6). Taken together, global analysis of the yeast secretome in the *PIL1* deletion strain revealed that 20.3% of secreted proteins, including a subset of secreted proteins with hydrolase activity, were more abundant in the *PIL1* deletion strain. The proportion of serine and threonine in the protein sequence of the hydrolases was particularly higher than the other identified proteins whereas in the sequence of signal peptides, the proportion of alanine is the highest in the more abundant protein cohort.

### *PIL1* deletion up-regulates the sphingolipid and serine biosynthesis pathways

**Fig. 6.**
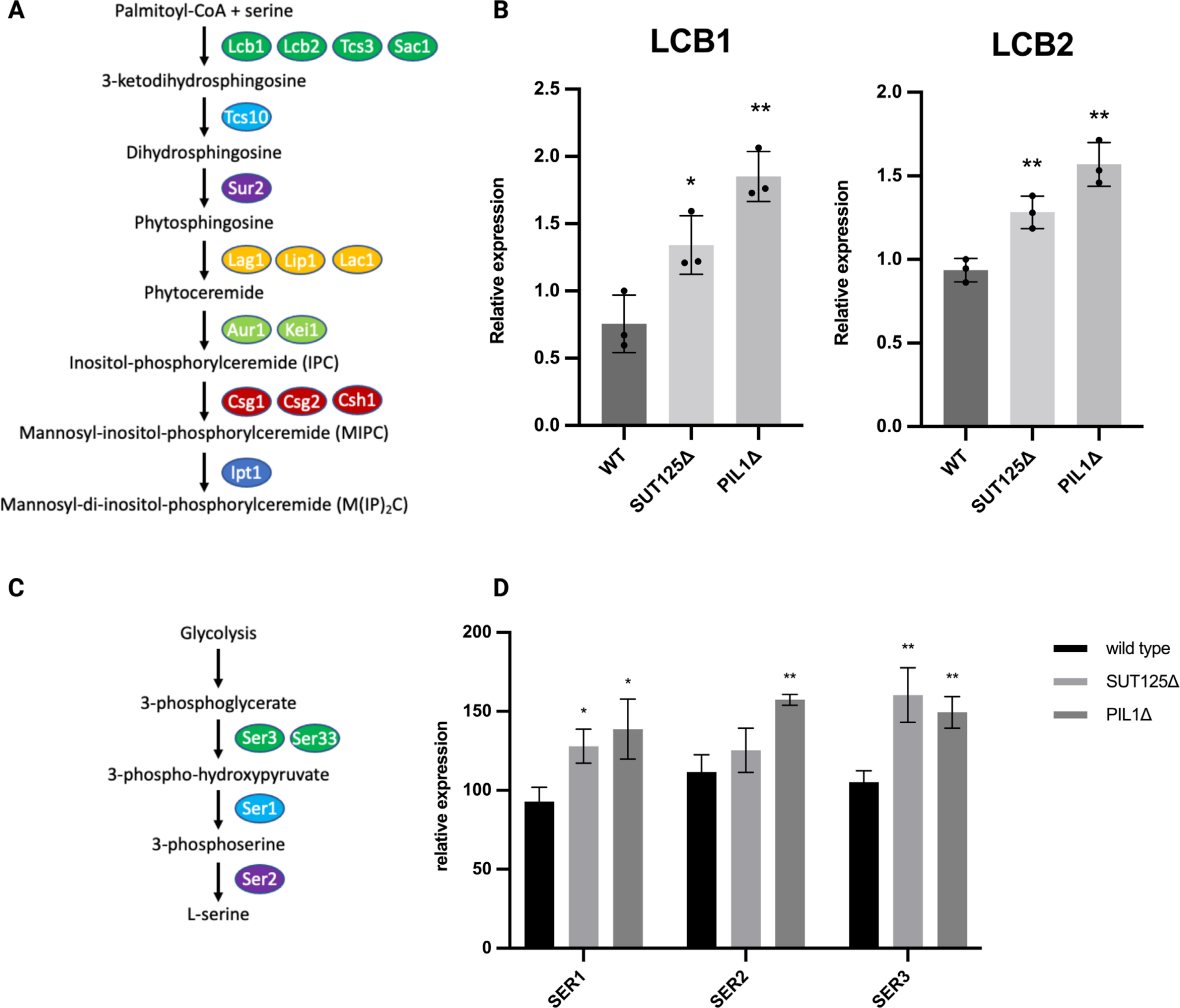
Up-regulation of sphingolipid pathway genes and serine synthesis pathway genes in *PIL1* deletion strain. (A) Sphingolipid biosynthesis pathway highlighting proteins required for each step. (B) Up-regulation of the first two key enzymes *LCB1* and *LCB2* in transcriptional levels following SUT125 and *PIL1* deletions. Unpaired Parametric t test was used. *P = 0.0296 and **P < 0.0075. (C) Serine biosynthesis pathway highlighting proteins required for each step. (D) Up-regulation of the three enzymes *SER1, SER2*, and *SER3* in transcriptional levels following SUT125 and *PIL1* deletions. Unpaired parametric t test was used. *P = 0.0196 and **P < 0.0023.

Eisosomes are enriched in sphingolipids, which are the essential components for vesicle formation and protein sorting in the secretory pathway. Therefore, we next set out to understand whether disrupting eisosomes had any effect on the sphingolipid biosynthesis pathway. The first two key enzymes of sphingolipid biosynthesis, *LCB1* and *LCB2*, are components of serine palmitoyltransferase, which is responsible for the condensation of serine with palmitoyl-CoA to form 3-ketosphinganine (Fig. 6A). Thus, *LCB1* and *LCB2* in wildtype, SUT125Δ and *PIL1*Δ strains were subjected to qPCR analysis. Intriguingly, the expression of both *LCB1* and *LCB2* were significantly increased in the SUT125Δ and further increased in the *PIL1*Δ strain when compared with the wild-type (Fig. 6B). In addition, the expression levels of other genes involved in sphingolipid biosynthesis were generally increased, especially when comparing the *PIL1*Δ with the wild-type (Figure S7). These results indicated that the sphingolipid biosynthesis pathway was up-regulated following *PIL1* deletion/eisosome disruption.

The biosynthesis of sphingolipids starts with the condensation of serine and palmitoyl-CoA in the endoplasmic reticulum. Interestingly, the global analysis of secretome in *PIL1* deletion revealed the proteins with a higher proportion of serine in amino acid composition had increased secretion. Therefore, we were interested in determining the effect on serine biosynthesis in the *PIL1*Δ strain. The three enzymes that are responsible for a three-step biochemical synthesis of serine: 3-phosphoglycerate dehydrogenase (encoded by *SER3*); phosphoserine aminotransferase (encoded by *SER1*); and phosphoserine phosphatase (encoded by *SER2*) were targeted for analysis (Fig. 6C). qPCR revealed increased expression of all three enzymes upon SUT125 and *PIL1* deletion (Fig. 6D), indicating an increase of the serine biosynthesis pathway, which may contribute to the synthesis of sphingolipids and the availability of serine in protein production.

## Discussion

In this study, we aimed to discover new targets to increase protein secretion in yeast by manipulating ncRNAs. A ncRNA deletion collection, including tRNAs, snRNAs, snoRNAs, SUTs, and CUTs was screened using an invertase assay, where the secretion of invertase was used as a proxy for protein secretion. In addition, the conventional invertase assay was optimized and performed in 96-well plate format to increase screening efficiency (49). Exploiting high-throughput screening, we identified several SUTs whose deletion increased invertase secretion. Only one of the identified SUTs (SUT390) is adjacent to a gene (APM1) with a known function in protein trafficking. However, the expression of APM1 was unchanged upon SUT390 deletion (Fig.   2A). Other identified ncRNA deletion strains with increased invertase secretion, SUT418 and SUT125, had no neighboring genes with any known direct relationship with protein secretion. It was not immediately clear why these ncRNA deletion strains increased invertase secretion. As ncRNAs regulate nearby gene expression (50–52), we examined the nearby gene expression of the identified SUTs. We found that in the SUT125 deletion strain down-regulation of *PIL1*, a gene encoding a core component of the eisosome/EMC, leads to the increased secretion of certain proteins, including heterologous proteins like antibodies. Eisosomes/EMCs were initially proposed to mark the static sites of endocytosis (24). However, the exact function of the eisosome remains controversial. Recent research has described the eisosome as a regulatory compartment for various cellular processes within the plasma membrane, including rapid membrane expansion in response to acute mechanical or osmotic stress, uptake of specific nutrients in response to starvation, and maintenance of membrane trafficking and sphingolipid signaling and biosynthesis (29, 45, 53, 54). Eisosomes/EMC consist of Pil1, Lsp1, Seg1, Sur7, and Nce102. Seg1 is located underneath the lipid bilayer and is recognized as a platform for the assembly of other eisosome components. Membrane curvature of the eisosome is determined by Seg1 abundance (55). Pil1 and Lsp1 contain a Bin1-amphiphysin-Rvs161/167 (BAR) domain. Pil1 forms a membrane-bound scaffold with Lsp1 through the binding of their positively charged BAR domains with negatively charged lipid bilayers to impose membrane curvature (56). Although Pil1 and Lsp1 share 72% sequence identity (24), the deletion of *LSP1* has no dramatic impact on eisosome formation, size or number, and only leads to mild defects in cellular signaling and endocytosis (57), as opposed to *PIL1* deletion (58). As *PIL1* and *LSP1* are equally expressed (Figure S8), we propose that the difference in function between Pil1 and Lsp1 in eisosomes is the underlying reason why *LSP1* deletion had no significant change in invertase secretion. Sur7 is one of the two transmembrane proteins in the eisosome and is proposed to initiate eisosome plasma membrane anchoring, as Sur7 is immobile and more stable than the other core components of the eisosome. The deletion of *SUR7* revealed mild defects in sporulation and modulation of sphingolipid content in the plasma membrane (59), but did not affect the localization of Lsp1 or Pil1 (24), indicating the eisosome was intact. Nce102, another transmembrane protein in the eisosome, is proposed to act as a sphingolipid sensor and communicates with other membrane compartments including MCPs and MCTs. Upon the disassembly or disruption of the eisosome, Nce102 shifts from sphingolipid-enriched areas to sphingolipid-depleted areas in the plasma membrane and approaches MCTs to activate the rapamycin-insensitive TOR complex 2 (TORC2) by Slm1/2 binding to trigger the Pkh1/2-Ypk1/2 sphingolipid signaling pathway (31, 34). Eisosomes are also enriched with sphingolipids (30, 31) and ergosterol (26, 27). Sustained sphingolipid biosynthesis is critical for the formation and function of eisosomes (53). Upon disruption of the eisosome by deleting *PIL1*, the sequestered sphingolipids and ergosterol are distributed more evenly within the plasma membrane (27).

Sphingolipids are important components for exocytosis/protein secretion. The Golgi network contains large amounts of sphingolipids, which are required to form secretory vesicles and regulate protein sorting (36, 37, 39, 40). Apart from the de novo formation of secretory vesicles, sphingolipids also modulate vesicle docking and priming as well as facilitating SNARE complex assembly, which is crucial for membrane fusion to activate vesicle exocytosis in *S. cerevisiae* and other species (60–62). In fact, the down-regulation of sphingolipid biosynthesis has been reported to lower the polarized localization of the exocyst, a conserved octameric complex that tethers secretory vesicles to the plasma membrane prior to membrane fusion (39, 63). Down-regulation of sphingolipid biosynthesis also reduced secretion of endoglucanase Bgl2 and endogenous invertase (39). Additionally, retention and accumulation of secretory vesicles approaching the plasma membrane was observed in a sphingolipid-deleted strain (39). Apart from yeast, lipid biosynthesis is important in the regulation of GPI-anchored protein transport and the maintenance of homeostasis in the early secretory pathway in human cells and plants, indicating the regulatory role of sphingolipids in the membrane trafficking and secretory pathways (35, 38, 64).

**Fig. 7.**
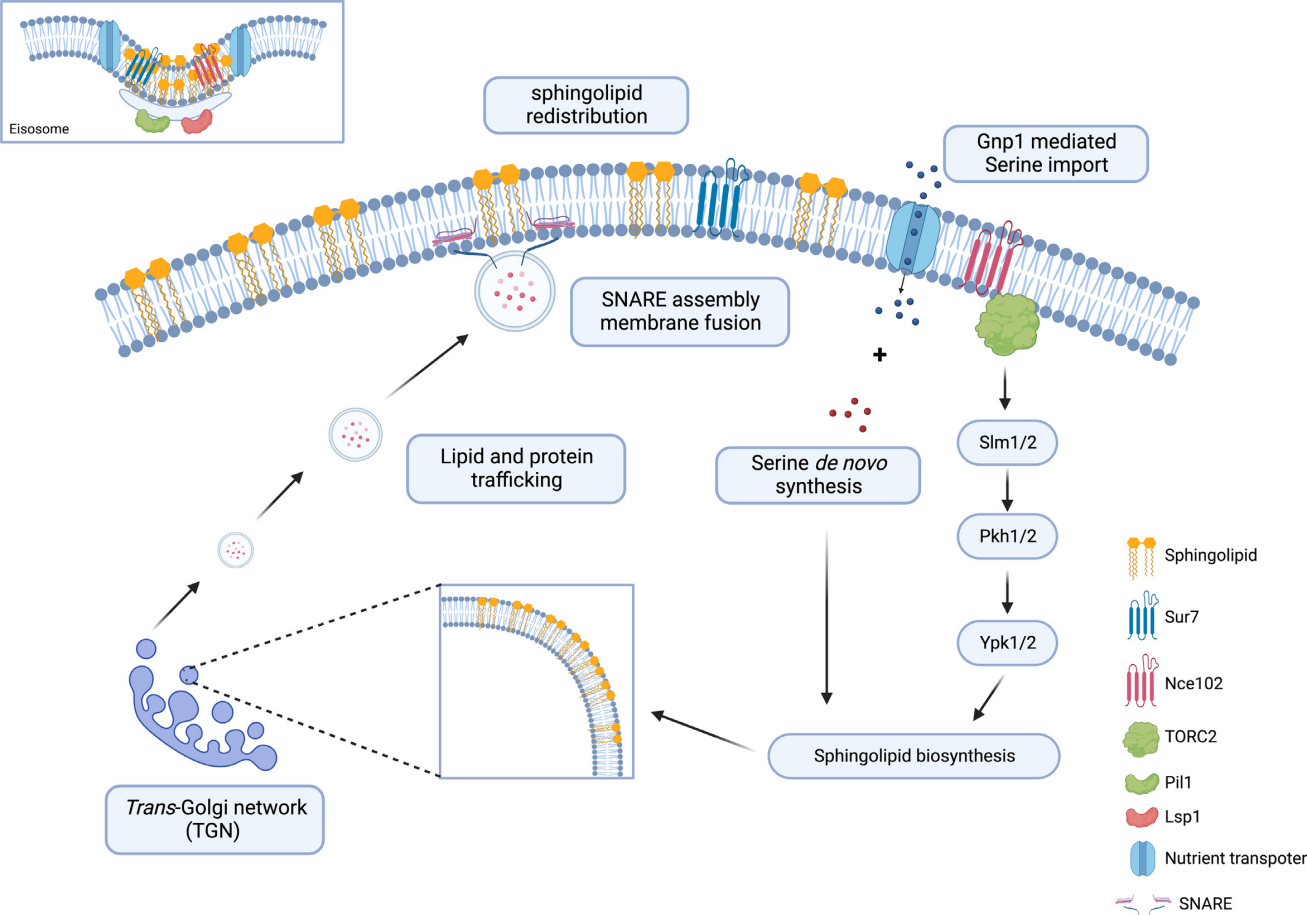
Eisosome disruption activates sphingolipid biosynthesis and exocytosis. (A) The eisosome integrity is maintained by Pil1 and Lsp1. Sphingolipids, APC transporters, and transmembrane proteins Sur7 and Nec102 are enriched in eisosomes. (B) By disrupting eisosomes, sphingolipids, APC transporters, and transmembrane proteins Sur7 and Nce102 are redistributed within the plasma membrane. (C) The binding of Nce102 to TORC2 in MCT domains in the sphingolipid-depleted area activates sphingolipid signaling and biosynthesis. (D) Serine de novo synthesis and Gnp1-mediated serine import further promote sphingolipid biosynthesis. (E) The increased efficiency of sphingolipid production contributes to the formation of secretory vesicles in the *trans*-Golgi network. (F) Sphingolipids redistributed in the plasma membrane also promotes SNARE assembly during membrane fusion.

We propose that the increase in protein secretion we observed following eisosome disruption is a result of redistribution of sphingolipids and the activation of sphingolipid biosynthesis. There are two possible mechanisms that may work together to increase exocytosis/protein secretion upon eisosome disruption. First, the activation of the sphingolipid biosynthesis pathway, resulting from Nce102 and Sur7 relocalization, may increase the amounts of sphingolipids in the *trans*-Golgi network, increasing vesicle formation and protein sorting in the secretory pathway. Second, the redistribution of large amounts of sphingolipids and ergosterol within the plasma membrane may provide more sites for the assembly of SNARE complexes that mark the sites of secretory vesicle docking and priming, and membrane fusion, thereby increasing the efficiency of membrane fusion and protein secretion (Fig. 7).

Global analysis in the yeast secretome revealed that *PIL1* deletion primarily increased the abundance of a subset of secreted proteins with hydrolase activity. The proportion of serine and threonine amino acid composition of these hydrolases, serine in particular, were higher than the other identified proteins (Fig. 5D). It has been reported that serine and threonine clustered in the protein sequence induced secretion of glucoamylase and galactosidase in yeast (65). However, the role of eisosomes in the secretion of these serine and threonine-enriched proteins has not yet been studied. *O*-linked glycans attached to the hydroxyl oxygen of serine and threonine may have an effect on protein secretion. However, only 12 o-glycosylated proteins were identified, implying that o-glycosylated proteins are not selectively affected in the *PIL1* deletion strain. Intriguingly, different from other lipids, sphingolipids use serine as the backbone for the attachment of acyl chains. The de novo sphingolipid biosynthesis begins with the condensation of serine and palmitoyl CoA by serine palmitoyltransferase. Thus, sphingolipid levels are tightly link to serine synthesis (66, 67). Apart from serine synthesis improving sphingolipid biosynthesis, exogenous serine uptake as the main source for sphingolipid biosynthesis has also been reported (68). The uptake of exogenous serine is mediated by Gnp1, a serine specific APC transporter. Intriguingly, APC transporters are sequestered in eisosomes in a stable and inactive status (69). In the presence of substrates, eisosomes were restructured while APC transporters were redistributed to the surrounding lipid domain where they imported the corresponding nutrients (69). Gnp1 may be activated upon eisosome disruption, leading to improved serine uptake and subsequent availability of serine. Here, by disrupting eisosomes, the availability of serine was increased either by de novo synthesis or environmental uptake. We propose that the increased availability of serine may promote the biosynthesis of sphingolipids (Fig. 7). Meanwhile, it may also improve the protein synthesis of serine-enriched secreted proteins. Regarding the influence of signal peptide, the pre-Ost1 performs the SRP-dependent cotranslational translocation, whereas the pre-pro-α-factor performs the SRP-independent post-translational translocation in yeast (70). During the SRP-dependent cotranslational translocation, the hydrophobic region of signal peptides is critical for efficient SRP recognition and protein translocation (71). Thus, the hydrophobicity of signal peptides may contribute to the efficiency of protein secretion. Analyzing the hydrophobic amino acids in the protein sequence of signal peptides, the higher abundance cohort had alanine as the predominant amino acid, which was also the major difference when compared with the lower abundance cohort (Figure S6). The higher composition of hydrophobic alanine indicated that the higher abundant protein cohort may be more likely to perform the SRP-dependent cotranslational translocation. In addition, since the increase of protein content in the higher abundance cohort (5.77%) was similar to the decrease of protein content in the lower abundance cohort (4.57%), meaning that the total protein content was increased by 1.2% that should not have detrimental effects for production of recombinant proteins (Table S2).

Taken together, we have found that ncRNA deletion influences the expression of eisosomes/EMC components leading to changes in sphingolipid signaling and biosynthesis. The disruption of eisosomes dramatically changes sphingolipid homeostasis, activating the sphingolipid biosynthesis pathway. The increased availability of sphingolipid, along with serine, the backbone of sphingolipid, may improve vesicle formation, membrane fusion, and synthesis of serine-enriched proteins, therefore, increasing exocytosis/protein secretion. These pathways may be new targets for increasing protein production in the industrially important yeast system.

## Materials and methods

### Strains and media

The ncRNA deletion collections were previously developed and stored in 96-well plates (19, 20). Only the coding region of each ncRNA was deleted without disrupting the promoter and terminator regions, or any adjacent genes. In the deletion collections, the BY4742 haploid strain (MATα his3Δ1 leu2Δ0 lys2Δ0 ura3Δ0) was used in this study (Table S3). Single and double deletion strains for *PIL1, LSP1*, and *SUR7* were confirmed by PCR analysis of genomic DNA from the respective deletion strains (Figure S2).

### Invertase assay

The deletion strains were duplicated to a new 96-deep-well plate and grown overnight. The overnight culture of each strain was normalized to OD_600_ of 0.5 and grown to their exponential phase in 1 ml YPD with 2% glucose (1% w/v yeast extract, 2% w/v Bacto-peptone, and 2% w/v glucose). Cells in the exponential phase were harvested and washed twice with distilled water. The cell pellets were resuspended in 1 ml YPD with 0.05% glucose (1% w/v yeast extract, 2% w/v bacto-peptone, and 0.05% w/v glucose) and incubated for 2 h. Cells were harvested and resuspended in distilled water. 1 × 10^6^ cells from each strain were transferred to a new 96-deep-well plate for the invertase assay.

A method for measuring invertase activity in *S. cerevisiae* was adapted for high throughput screening of invertase secretion in the deletion collection of ncRNA (49). To start the reactions, 50 μL of 50 mM sodium acetate pH5.1 was added to each cell sample. Next, 12.5 μL of fresh 0.5 M sucrose was added to each sample except for the sucrose control. After a quick spin, the 96-deep-well plate was incubated at 37°C for 10 min. The plate was then put on ice and 75 μL of 0.2 M K_2_HPO_4_ pH 7.0 was added. To stop the reaction, the plate was heated to 100°C for 3 min and then cooled down on ice. A volume of 500 μL of assay mix was freshly made and added to each sample (assay mix: 50 μL glucose oxidase 5000 U/mL; 62.5 μL peroxidase 1 mg/mL; 375 μL o-dianisidine 10 mg/mL; brought to 25 mL with K_2_HPO_4_ 0.1 M pH7.0). The plate was incubated at 37°C for 10 min. A volume of 500 μL of 6 N HCl was added to each sample. The changes of color were measured at OD_540_ using a microplate reader.

### Reverse transcription qPCR

Total RNA was extracted using the RiboPure Yeast Kit (Life Technologies) and converted to cDNAs using SuperScript IV Reverse Transcriptase (Life Technologies). SYBR Green Master Mix was added to cDNA samples according to the manufacturer’s instructions (Applied Biosystems). Forward and reverse primers were designed to generate 100 to 300 bp amplicons (Table S4). The primer specificity was checked by conventional PCR and electrophoresis. At least three biological and technical replicates were made for each sample. At least two control genes including *UBC6, TFC1, TAF10​*, and *​TUM1​* were used for normalization. The run method was set as follows: 95°C for 10 min (holding stage); 95°C for 15 s, 60°C for 1 min, 40 cycles (cycling stage). Melting curve was included when new primers were used. Following the holding and cycling stages, the melting curve stage was set as follows: 95°C for 15 s; 60°C for 1 min (+ 0.3°C/cycle to 95°C).

### Expression plasmid construction

The GFP expression cassettes in plasmids YIplac204TC-pre-Ost1-msGFP and YIplac204TC-pre-pro-aFactor-msGFP were amplified with two primers that add the restriction enzyme sites XhoI and EcoRV to each end (48) (Table S4). Both expression cassettes and the vector pRS403 were digested with XhoI (NEB, catalogue number R0146S) and EcoRV (NEB, catalogue number R3195S) restriction enzymes at 37°C for 1 h. The digested plasmid was treated with 1 μL alkaline phosphatase, calf intestinal (CIP, NEB, catalogue number M0290) at 37°C for 15 min. All digestions were purified by GenElute PCR Clean-Up Kit (Sigma, catalogue number NA1020). The digested pre-Ost1-GFP and pre-pro-α Factor-GFP expression cassettes were cloned in pRS403 plasmid by T4 DNA ligation (NEB, catalogue number M0202S). The Pex expression plasmid was constructed by Gibson cloning into pRS403 plasmid according to the manufacturer’s instructions (Gibson Assembly Cloning Kit, Cat# E5510S; Table S4).

### Yeast transformation and collection of secreted proteins

The reconstructed expression plasmid pRS403-pre-Ost1-GFP, pRS403-pre-pro-α Factor-GFP, and pRS403-pre-Ost1-Pex were linearized by NdeI and transformed into BY4742 wild-type or *PIL1* mutant strains. Transformants were selected on SD-His plates and single colonies were cultured overnight in SD-His media. Cultures were normalized to OD_600_ of 0.5 in 200 mL SD-His medium and cultured for 4 h. The culture was centrifuged at 500 × *g* for 10 min. Supernatant was transferred to a new container and centrifuged at 1,000 × *g* for 10 min, followed by another centrifugation in a new container at 5,000 × *g* for 10 min. All centrifugations were performed with slow acceleration and deceleration. Supernatant was concentrated using a 10 K cutoff filter device (Amicon Ultra-15 10 K) to a final volume of 200 μL. Culture medium was exchanged by adding distilled water to the filter device. A volume of 10 μL of enriched supernatant was mixed with SDS-PAGE loading dye, heated to 95°C and run on 12% SDS-PAGE at 200 V for 1 h.

### Western blotting

SDS-PAGE gel was electrotransferred (BioRad TransBlot) to nitrocellulose membrane and incubated with primary antibody (for GFP, mouse anti-GFP, 1:1,000, catalogue number G6539, Sigma Merck), (for Pex, mouse anti-6xHis, 1:1,000, ThermoFisher, catalogue number MA1-21315) then peroxidase-conjugated secondary antibody (rabbit antimouse, 1:5,000, catalogue number A9044 Sigma Merck). Chemiluminescent detection was then carried out according to the manufacturer’s instructions (Millipore Immobilon Western WBKLS0050). The membrane was scanned in a LI-COR machine (LI-COR Odyssey Fc) in the chemiluminescent channel for 10 min.

### SILAC

Mutant and wild-type strains were inoculated into 5 mL of SD-Lys-Arg medium, supplemented with 50 mg/L L-Lysine-3,3,4,4,5,5,6,6-d8 hydrochloride (Lys8; catalogue number 616214, Sigma Merck) and 50 mg/L L-Arginine-^13^C_6_ hydrochloride (Arg6; catalogue number 643440, Sigma Merck) for the mutant or 50 mg/L L-Lysine hydrochloride (Lys0) and 50 mg/L L-Arginine hydrochloride (Arg0) for the wild-type and cultured overnight at 30°C. Overnight cultures were normalized to OD_600_ of 0.5 with 25 mL of SD-Lys-Arg media, supplemented with 50 mg/L Lys8, Arg6 for the mutant or Lys0, Arg0 for the wild-type, and incubated at 30°C for 4 h. The cultures of mutant and wild-type were combined and centrifuged at 500 × *g* for 15 min. Supernatant was carefully transferred to a new centrifuge bottle and centrifuged again at 1,000 × *g* for 10 min. The supernatant was then transferred to another new bottle and centrifuged at 5,000 × *g* for 10 min. These sequential centrifugations were performed with low acceleration and deceleration to minimize cell damage and the release of cell contents. The final supernatant was concentrated with a 10 kDa filter (Amicon Ultra-15 10 K) by multiple loading and centrifugation at 4,000 × *g* for 10 min until all the volume was loaded. Centrifugation was carried out at 4,000 × *g* for 30 min to further concentrate the protein samples. Buffer exchange was carried out by adding 15 mL 1X S-Trap lysis buffer (5% SDS with 50 mM Triethylammonium bicarbonate (TEAB, Thermo Scientific catalogue number 90114; pH 7.5) to the filter followed by centrifugation at 4,000 × *g* for 30 min. The 150 to 200 μL final concentrated protein was reduced by adding Dithiothreitol (DTT) to a final concentration of 10 mM followed by incubation at 60°C for 10 min to reduce cysteine bonds. To modify free cysteine and stabilize the protein, iodoacetamide (IAM) was added to a final concentration of 15 mM. The sample was then placed in the dark for 30 min. To quench any free IAM, the same amount of DTT was added as before. S-Trap Micro Spin Column Digestion (PROTIFI) was carried out using 20 μg of Trypsin to digest the protein at 47°C for 1 h. The digested peptides were desalted by OLIGO R3 Reversed-Phase Resin (Thermo Fisher Scientific) and finally eluted in 100 μL of 0.1% formic acid in 30% acetonitrile. Samples were run on the Q Exactive HF Orbitrap LC–MS/MS System (Thermo Fisher Scientific) with 90 min acquisition time. Data were analyzed by Proteome Discoverer Software (Thermo Fisher Scientific).

### Gene ontology analysis

The gene ontology enrichment analysis was conducted by using the gene ontology knowledgebase (http://geneontology.org/). The genes encoding secretory proteins with up- or down-regulated abundance were enriched relative to the whole genome of *S. cerevisiae*.

## Supplementary Material

pgac241_Supplemental_Files

## Data Availability

All study data are included in the article and/or supporting information.
