# Supplementary material for: Eisosome disruption by noncoding RNA deletion increases protein secretion in yeast
Source: PNAS Nexus. 2022 Oct 26;1(5):pgac241. doi: 10.1093/pnasnexus/pgac241 (PMC9802208; doi:10.1093/pnasnexus/pgac241)
Supplement: pgac241_Supplemental_Files [file pgac241_supplemental_files.zip › PNASNEXUS-PNASNEXUS-2021-00011-T-s01.pdf]

Supplementary Figures

Eisosome disruption by non-coding RNA deletion increases protein secretion in yeast

Matthew Wenjie Feng[1], Daniela Delneri[1,2], Catherine B Millar[1], Raymond T O'Keefe[1*]

[1]Division of Evolution, Infection and Genomics, Faculty of Biology, Medicine and Health, The University of Manchester, Oxford Road, Manchester M13 9PL, UK

[2]Manchester Institute of Biotechnology, Faculty of Biology Medicine and Health, The University of Manchester, 131 Princess street, M1 7DN, UK

*Correspondence should be addressed to Raymond T O'Keefe (rokeefe@manchester.ac.uk)

Key words: non-coding RNA, PIL1, eisosome membrane compartment (EMC), sphingolipids, protein secretion, *Saccharomyces cerevisiae*

A

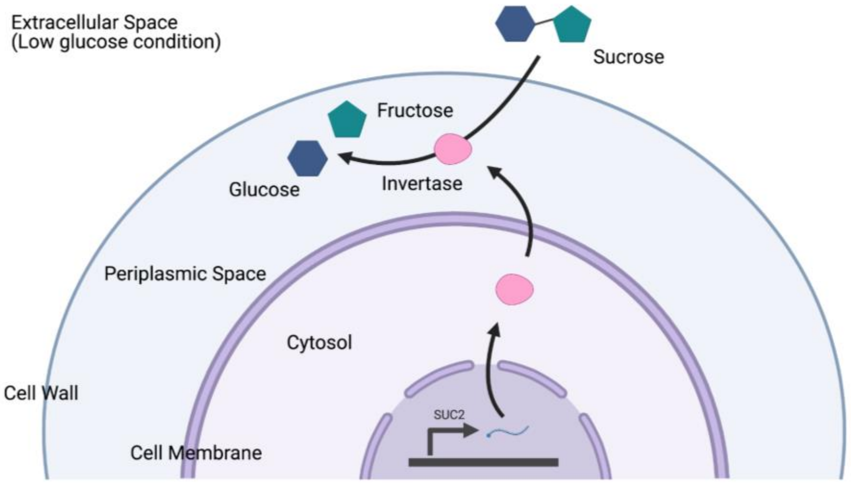

B

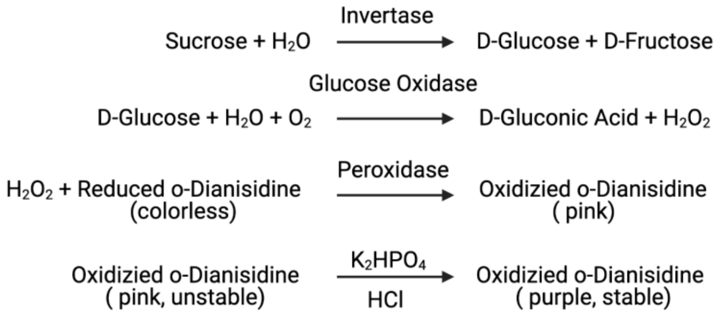

Fig.S1 The principle of the invertase assay and colorimetric reaction. A. The *SUC2* gene is activated to produce invertase by culturing with low glucose (0.05%). The resulting invertase is secreted into the periplasmic space. The secreted invertase converts extracellular sucrose into glucose that can be taken up by the cell. **B.** Secreted invertase activity is quantified based on the glucose produced by invertase. The glucose produced by invertase is oxidised by glucose oxidase in the presence of oxygen and water to produce hydrogen peroxide. The reduced o-Dianisidine (colorless) is oxidised by peroxidase in the presence of hydrogen peroxide to produce oxidized o-Dianisidine (pink) and further stabilised (purple) in an acidic environment.

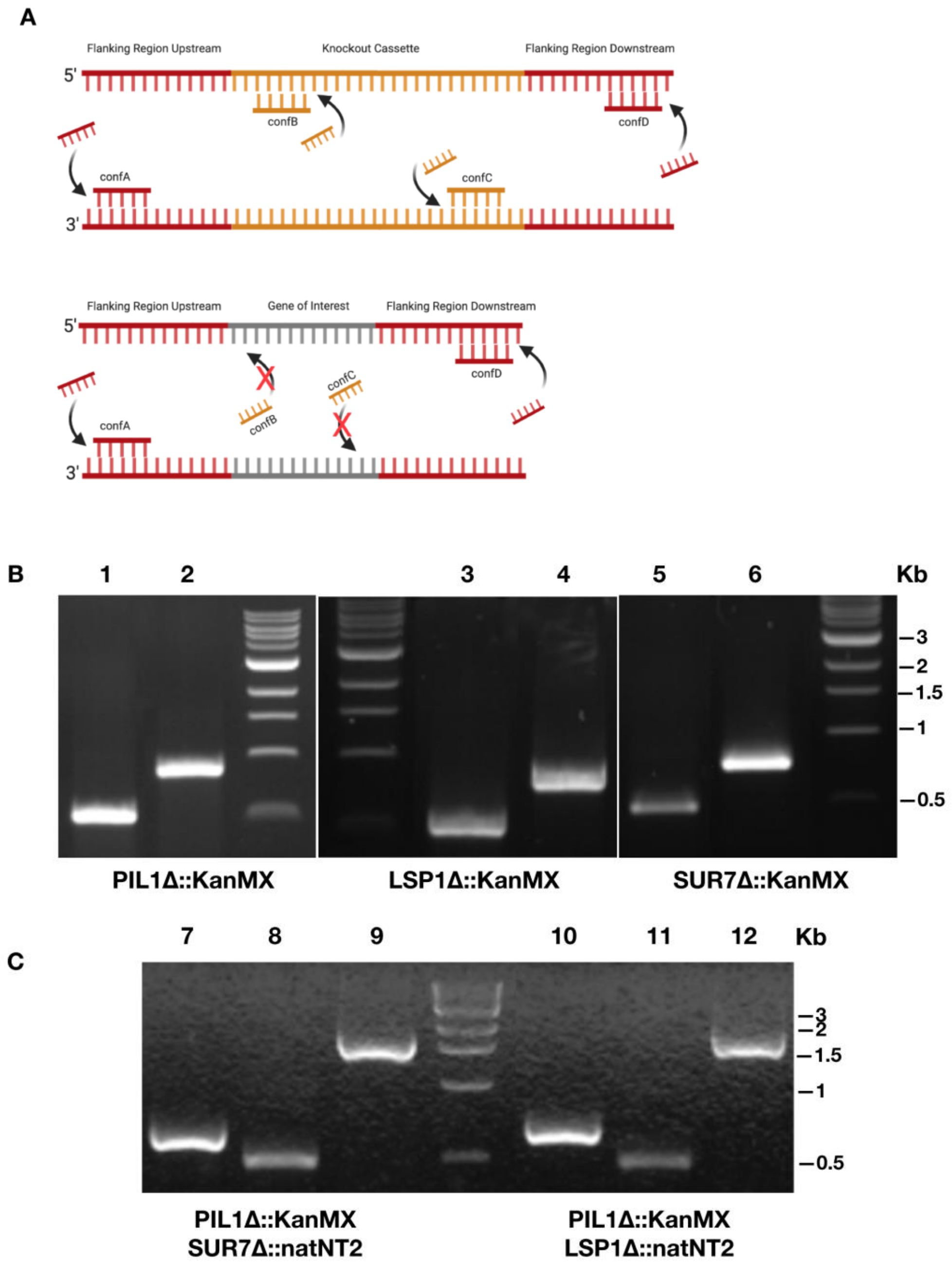

Fig.S2 Confirmation of deletion strains.

A. Illustration of confirmation PCR strategy. **B.** Single deletion of PIL1, LSP1 and SUR7. Lanes 1, 3 and 5 display amplification of the 5' junction across the upstream region of PIL1, LSP1 and SUR7 and the region of KanMX knockout cassette using primers confA and confB. Lanes 2, 4 and 6 display amplification of the 3' junction across the downstream region of PIL1, LSP1 and SUR7 and the region of KanMX knockout cassette using primers confC and confD. The presence of bands indicates a successful knock-in of KanMX cassette and knock-out of the corresponding genes. **C.** Double deletion of PIL1/SUR7 and PIL1/LSP1. Lanes 7 and 10 display amplification of the 5' junction across the upstream region of SUR7 and LSP1 and the region of natNT2 knockout cassette using primers confA and confB. Lanes 8 and 11 display amplification of the 3' junction across the downstream region of SUR7 and LSP1 and the region of natNT2 knockout cassette using primers confC and confD. The presence of bands indicated a successful knock-in of natNT2 cassette and a knock-out of the corresponding genes. Lanes 9 and 12 display amplification of the entire genomic region of homologous recombination using primers confA and

confD. The size of bands indicates a successful homologous recombination has taken place to replace the corresponding gene with natNT2 cassette.

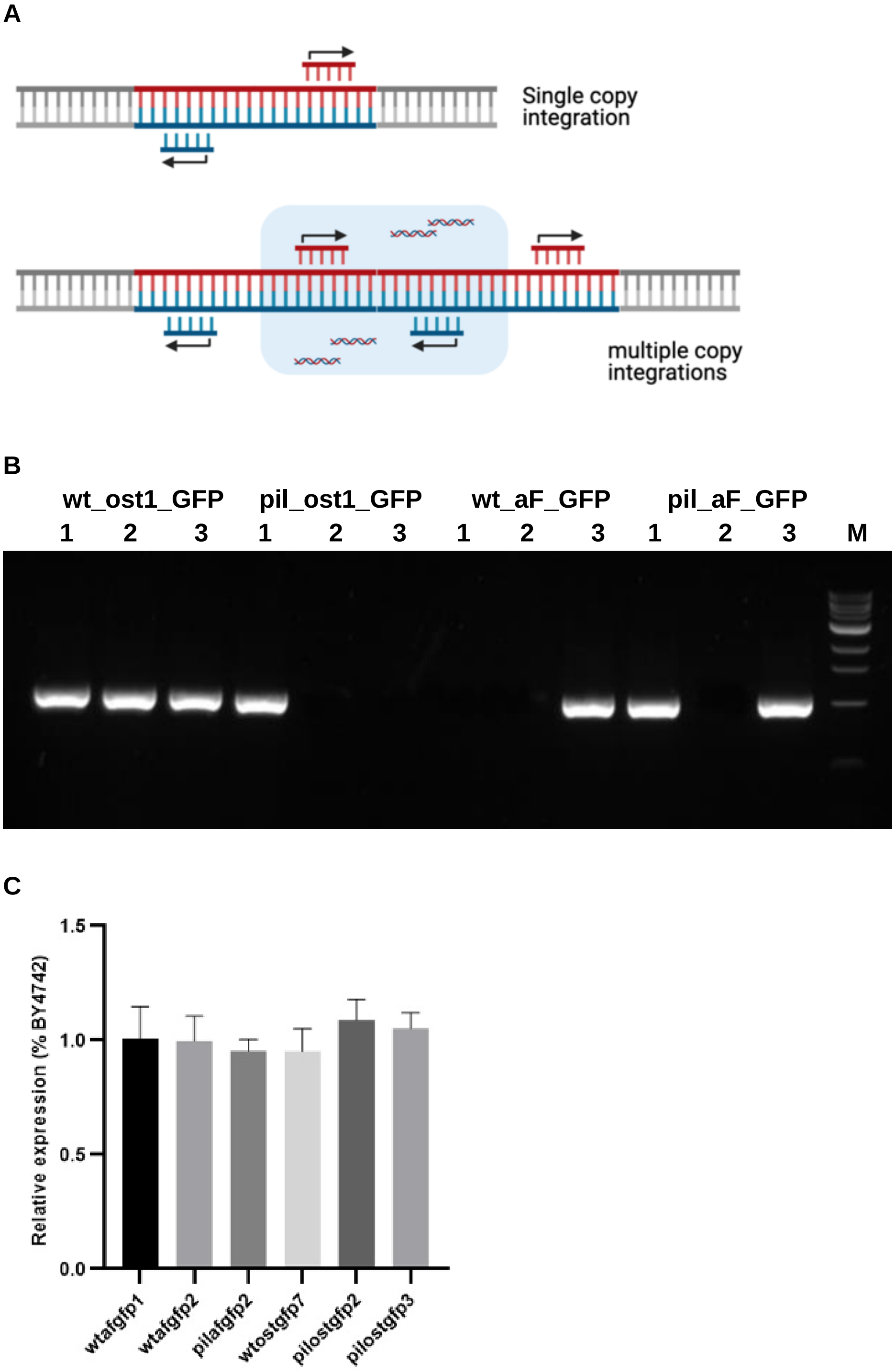

Fig.S3 Confirmation of pre-Ost1-GFP and pre-pro-α Factor-GFP copy number integrated into the genome of wild type and *PIL1* deletion strains. A. The principle of diagnostic PCR. The designed primers are only able to amplify in the presence of multiple copy integrations. **B.** Three single colonies of the PIL1 deletion strain and the wild type strain integrated with pre-Ost1-GFP and pre-pro-α Factor-GFP expression cassettes, respectively, were examined. Lanes with bands indicated multiple copy integrations were present, whereas blank lanes are indications of single copy integration. **C.** The mRNA levels of cells with single copy GFP. qPCR showed no significant difference among them

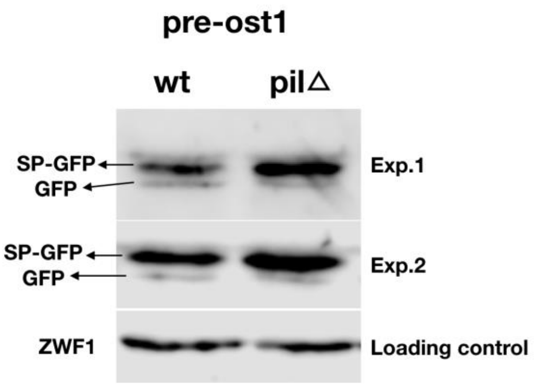

Fig.S4 Intracellular GFP levels linked with pre-ost1 signal peptide in the wild type and *PIL1* deletion strains. SP-GFP: GFP precursor linked with a signal peptide; GFP: matured protein; ZWF1: endogenous loading control. Two independent experiments (Exp.1 and Exp.2) were carried out.

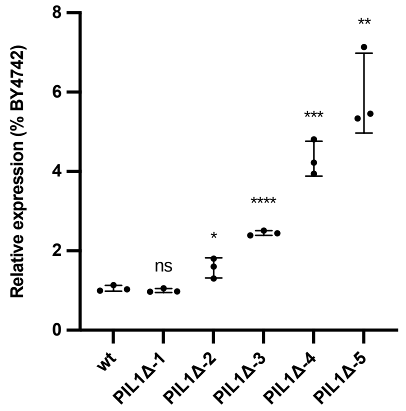

Fig.S5 Confirmation of pre-Ost1-Pex mRNA levels in the genome of wild type and *PIL1* deletion strains. *PIL1*Δ1-5 represent different colonies expressing Pex protein in various levels. The qPCR in *PIL1*Δ-1 revealed no significant difference in Pex expression when compared with the wild type, whereas *PIL1*Δ2-5 displayed higher expression levels of Pex. Unpaired Parametric T-test was used. ****p <0.0001 ***p=0.0002 **p=0.0011 *p=0.0273.

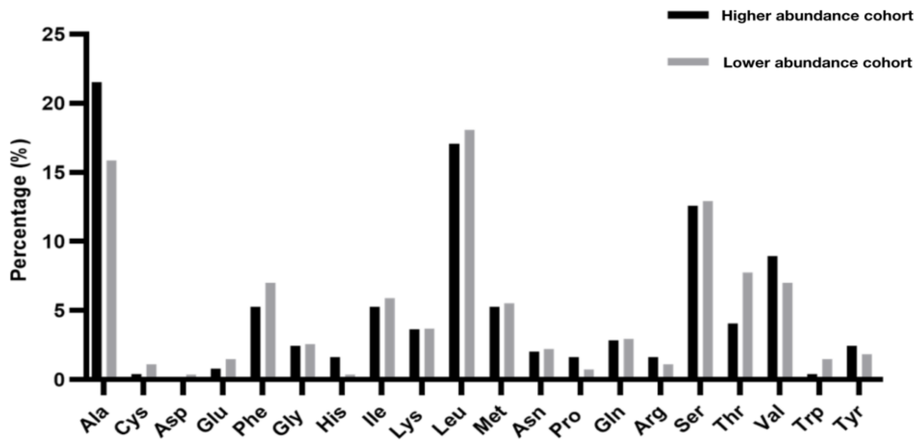

Fig.S6 Percentages of amino acids in signal peptide sequences of secreted proteins identified with higher or lower abundance by LC-MS/MS. Data were derived from three SILAC experiments.

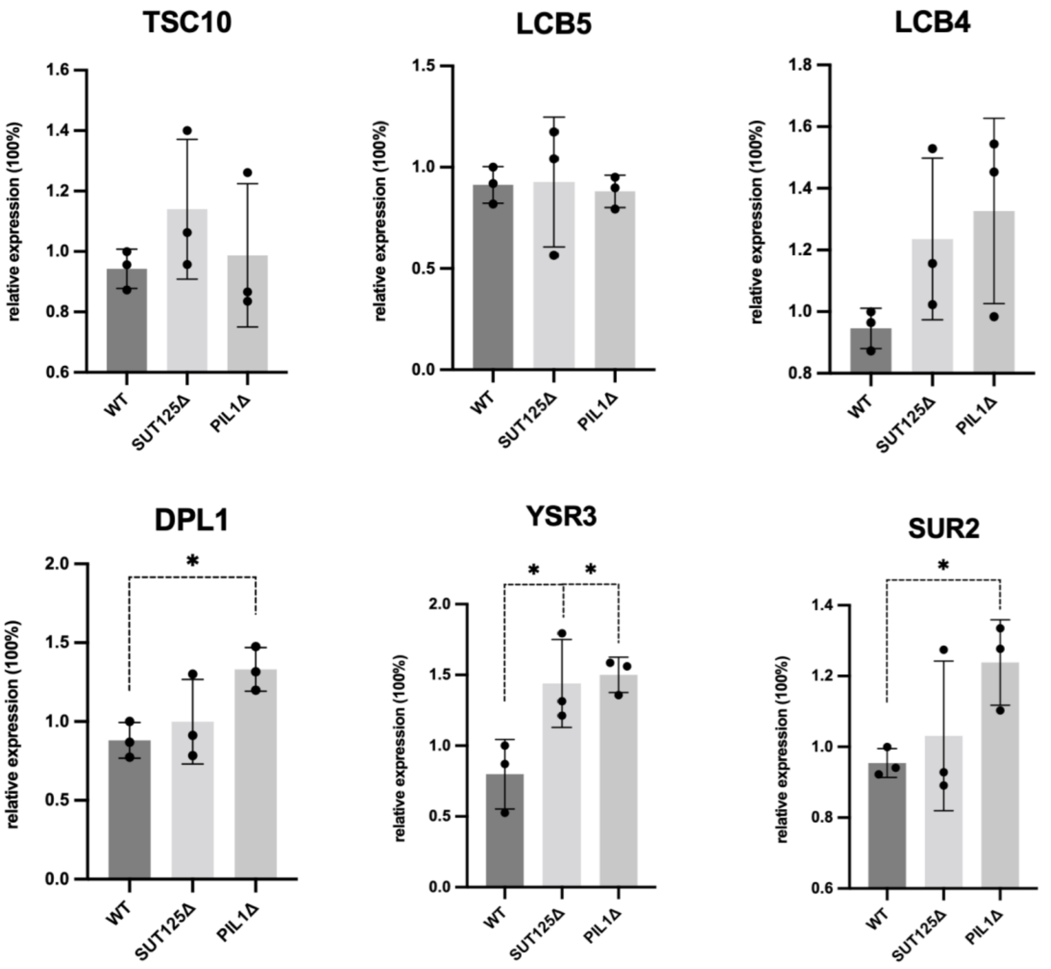

Fig.S7 Changes in expression levels of other genes involved in sphingolipid biosynthesis. Unpaired Parametric T-test was used. *p<0.05

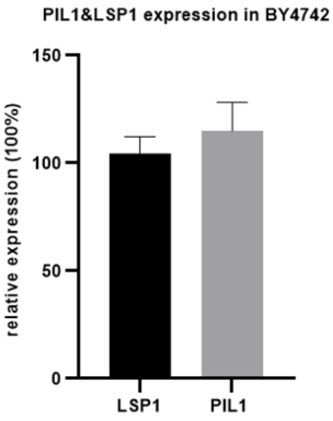

Fig.S8 The mRNA levels of PIL1 and LSP1 in the BY4742 wild type strain. No significant differences were observed between the gene expression of *PIL1* and *LSP1*.
